# Factors influencing contraceptive use among women of advanced reproductive age in Nigeria

**DOI:** 10.1186/s40834-025-00412-0

**Published:** 2025-11-27

**Authors:** Ifedapo Agbeja, Funmilola Folasade Oyinlola, Omolayo Bukola Oluwatope, Immanuel Shittu, Ibukun Agbeja, Sukurah Adewumi Hammed, Bukola Beatrice Howells

**Affiliations:** 1 Department of Demography and Social Statistics, Faculty of Social Sciences, Obafemi Awolowo University, Ile-Ife, Osun State Nigeria; 2 The Khana Group, Abuja, Nigeria; 3 National Centre for Technology Management, Obafemi Awolowo University, Ile- Ife, Nigeria; 4 Ogun State College of Nursing Sciences, Abeokuta, Ogun State Nigeria

**Keywords:** Contraceptive use, Advanced reproductive age, Unmet need, Relative risk, Nigeria

## Abstract

**Background:**

Contraceptive adoption is crucial for reducing unplanned pregnancies and maternal mortality and improving reproductive health outcomes. However, the unmet need for family planning remains high in developing countries. This study examines the prevalence and factors influencing contraceptive use and non-use among women of advanced reproductive age (35–49) in Nigeria.

**Methods:**

This was a cross-sectional study of 11,248 women of advanced reproductive age (35–49 years), from the most recent Nigeria Multiple Indicator Cluster Survey (MICS). This study utilised secondary data and information on socio-demographic characteristics, reproductive history, and contraceptive use. We employed a multinomial logistic regression in examining the bivariate relationship between the use and non-use of contraceptives and explanatory variables. Relative risk ratios (RRR) were calculated to indicate influence of the selected socio-demographic variables on the use and non-use of contraceptives. Analysis was performed using Stata version 17.

**Results:**

The prevalence of contraceptive non-adoption among women of advanced reproductive age was 71%. Among those who use contraceptives, 21% practice modern methods, and 7% rely on traditional methods. Factors such as ethnicity, region, marital status, media exposure and experience of child mortality all had a significant positive association with contraceptive adoption rate. Additionally, a significant positive association was found between age, education, parity, wealth index, fertility desire and contraceptive use. At the multivariate level, education (RRR = 1.49; *p* = 0.050), ethnicity (RRR = 0.298; *p* = 0.001), region (RRR = 4.173; *p* < 0.001), and wealth status (RRR = 0.568; *p* = 0.002) were significantly associated with contraceptive non-use.

**Conclusions:**

The study highlighted a high prevalence of contraceptive non-adoption among women of advanced reproductive age, with more than two-thirds not using any method of family planning. Socio-demographic factors, importantly, maternal education consistently emerged as a strong predictor of contraceptive use, underscoring the critical role of female empowerment and access to education in improving reproductive health outcomes. These findings suggest the need for targeted interventions that address regional and socio-cultural disparities, strengthen health education, and promote equitable access to family planning services to reduce unmet needs and enhance reproductive health among older women in Nigeria.

**Clinical trial number:**

Not applicable.

## Introduction

The world population in 2015 was approximately 7.3 billion; by 2023, it had grown to around 8.5 billion, and it is estimated to reach 9 billion by 2037. This high growth rate is due to the lack of adoption and utilization of adequate contraceptives, particularly the modern contraceptive such as condoms, pills, implants, injections, intrauterine devices (IUDs), and sterilization which are scientifically proven methods for preventing pregnancy. This is because the adoption of contraceptives plays an important role in controlling rapid population growth and reducing infant and maternal mortality and morbidity. However, the level of fertility also plays a significant role, with countries like Nigeria having high fertility rates. As of 2016, Nigerian women had an average of 5.53 children each, contributing to the nation’s population of over 200 million, which is projected to increase by 44% by 2030. Although if the full demand for contraceptives were met, it is estimated that 67 million unintended pregnancies could be prevented [1].

As of 2021, it was estimated that there were 1.9 billion women in the reproductive years (15–49), 58% (1.1 billion) of these women have unmet needs for modern contraceptives, while only 842 million of the women in their reproductive years adopted the use of modern contraceptive methods. Similarly, women in low and middle income countries, estimated at about 214 million, who wanted to use contraceptives to avoid pregnancy, were not using them. This lack of conceptive adoption among these women has contributed to the increase in their maternal mortality [2, 3]. Recent studies have shown that the adoption of contraceptives, specifically modern contraceptives, could lead to a significant reduction in maternal mortality from 308,000 to 84,000 and newborn mortality from 2.7 million to 538,000 per year, respectively [2]. 

Globally, there has been a significant increase in the demand for the use of modern contraceptives, particularly in the developed world. However, despite its benefits, the acceptability rate remains low in developing countries, especially in Africa, which experienced only an increase of 4.6% in modern contraceptive usage among married women and those in relationships between 2012 and 2017, with no noticeable increase in West Africa, which is made up of developing countries like Nigeria [4–6]. As of 2012, developing countries accounted for 73% of the global unmet needs (that is, females in their reproductive age who wish to avoid pregnancy but are not using contraceptives), with an increment of about 9 million (from 153 million to 162 million) between 2008 and 2012 before hitting 214 million in 2023 [5]. Although there are limited studies that focus on advanced reproductive ages (35–49), evidence indicates that about 23 million adolescent girls aged 15 to 19 in developing countries have unmet needs for contraceptive adoption. While those of the advanced reproductive age are not accounted for [7].

Interestingly, it has been projected that contraceptive use will increase across various regions of Sub-Saharan Africa (SSA) by 2030. For instance, in West Africa, contraceptive usage is expected to increase by 10% from 17% to 27%, while in Eastern Africa, it is expected to increase by 15% from 40% to 55%, and in Middle Africa, it is expected to increase by 11% from 23% to 34%. However, even with these projected increases, it is unlikely that the Sub-Saharan Africa (SSA) region will reduce the projected levels of unmet needs in the region [8]. 

In Nigeria, like most developing countries, contraceptive utilization among women of reproductive age, especially those of advanced reproductive age (35–49), is still low. The most recent National Demographic and Household Survey (NDHS) revealed that there is a 17% contraceptive prevalence rate among married women who are between the ages of 15 and 49. Among sexually active unmarried women within the same age group (15–49), only 37% are reported to adopt contraceptive use [1]. Despite the awareness of contraceptive use at health centers, the actual utilization of contraceptives to prevent unintended pregnancies remains low in the country, especially in the northern region, which has the lowest rates of contraceptive use. Previous studies have shown that unexpected or unplanned pregnancies, unsafe abortions, neonatal deaths, and maternal mortality are associated with low and non-contraceptive use, which is driven by various factors [3].

In developing countries like Nigeria, several factors have been found to influence the non-use of contraceptives among women of reproductive age, some of which include the socioeconomic status of the women, access to quality contraceptive services, concerns regarding the side effects of contraceptive methods (such as weight loss or gain, heavy bleeding, and lack of libido), approval from their male partners, educational status, religious belief, and cultural belief among others [2, 4, 9, 10].

However, several studies have been carried out to understand the prevalence and factors influencing contraceptive use and non-use among women of reproductive age. For instance, a study conducted by [5] to understand “Trends and Determinants of Non-Utilization of Modern Contraception in Ekiti State, Nigeria” revealed that the unmet need for contraceptives declined by 9.4% between 2008 (84.8%) and 2018 (75.4%) as a result of increased contraceptive awareness and further proposed that proper education against barriers of carefree attitudes, religious prohibition, misconceptions, fears, myths, and hearsay can help to reduce the non-use of contraceptive.

In another study conducted by [4] to understand the “Prevalence and factors associated with modern contraceptive use among women of reproductive age in 20 African countries,” it was revealed that the prevalence of contraceptives in Africa was generally low. However, Injectable contraceptives were the most preferred contraceptive method, followed by oral pills and implants. The study also found that factors associated with a higher likelihood of using contraceptives among women of reproductive age included having an education, attending two or more antenatal care visits, being aged 25 to 34, and having a middle- or high-income status. Conversely, women living in rural areas, those who were never married, those without access to media, and nulliparous women (women who have never given birth) were less likely to use any contraceptive methods.

Furthermore, a study conducted by Ezenwaka et al. 2020(author’s name is missing) [7] aimed at “Exploring factors constraining utilization of contraceptive services among adolescents in Southeast Nigeria” revealed that poor knowledge of contraceptive methods, lack of boldness to seek contraceptive services when needed, and fear of side effects of contraceptives were the top factors influencing the non-use of contraceptives. While these studies provide valuable insights into the prevalence and factors influencing contraceptive use and non-use among women of reproductive age in Nigeria, they typically examine all ages (15–49) without paying specific attention to the dynamics of contraceptive use and non-use among women of advanced reproductive age (35–49). Moreover, these studies sometimes use qualitative primary sources to get information. In addition, most utilised the National Demographic and Health Survey (NDHS) and overlooked other relevant Nigerian data sources beyond the NDHS.

This study aims to address the limitations of previous research and enhance the existing body of knowledge by examining the prevalence and factors influencing contraceptive use and non-use among women of advanced reproductive age (35–49) in Nigeria. Women in this age group deserve special attention in demographic and health research for several significant reasons. They are at a higher risk of experiencing high-risk pregnancies, which can lead to adverse maternal and perinatal outcomes [11]. Additionally, effective contraceptive use can help prevent unintended pregnancies among all women, including those in this age range. However, it is essential to recognize that some women of advanced reproductive age may not have completed their families. Some may still be childless, while others may have only begun having children or have chosen to delay childbearing to pursue personal goals. Therefore, understanding their unique circumstances is essential [11]. Furthermore, this study will utilize data from the Multiple Indicator Cluster Survey (MICS), a more recent source of demographic data.

## Methods

### Study setting and data source

The geographic domain of the study is Nigeria, West Africa. The population of Nigeria is currently estimated at over 200 million people [12]. The birth rate is high in the country, with a total fertility rate of over five children per woman. The death rate is lower, resulting in a high natural increase. Infant and maternal mortality rates in Nigeria were among the highest in West Africa. Contraceptive prevalence rates, either for all methods or for modern methods, were less than 12% in the country [13]. However, population and health policies are being implemented to improve population health in the country. The key population policy is the 2022 Revised National Population Policy on population for sustainable development. The policy seeks to improve maternal, newborn, child, adolescent, reproductive, and elderly health, promoting voluntary fertility regulation and achieving a moderate population growth rate [14]. The policy also emphasizes respect for the rights of individuals and couples in shaping the quality of their lives and their wellbeing.

This study employed a secondary data of the Multiple Indicator Cluster Survey (MICS) 2021 with a nationally representative household sample of 38,806 households residing in non-institutional dwelling units in rural and urban Nigeria. The list of enumeration areas (EAs) for the 2006 census, updated in February 2021, was the sampling frame of the 2021 MICS survey. The primary sampling units (PSUs), which were considered clusters in the 2021 MICS, were defined based on Enumeration Areas (EAs) from the 2006 census EA frames [15]. A total of 11,248 women of advanced reproductive age and ever-given birth were eligible and interviewed for this study. Thus, the data on women was obtained and analysed from the Multiple Indicator Cluster Survey dataset. The survey provided estimates of the key health indicators at the national and regional levels.

### Variables of the study

#### Outcome variable

The outcome variable was current contraceptive use, which has three possible categories, namely non-use [1], using a traditional method [2], and using a modern method [3, 11, 16]. All women who reported non-use of any method were grouped as “non-use” while women who reported using any modern method, such as sterilizations, condoms, injectables, implants, pills, diaphragms, and Foam/jelly, were grouped as “using modern method”. Women who reported the use of traditional methods such as Lactation Amenorrhea, Abstinence, and withdrawal were grouped as “using traditional methods”.

#### Explanatory variables

The variables used in this study were selected because of their association with the outcome variables from previous studies [11, 17] as well as their availability in the MICS Dataset. To validate the extent of association to which the independent variables influence the contraceptive use among women of advanced reproductive age in Nigeria, the independent variables were grouped into socio-demographic factors (such as age, highest level of education, marital status, place of residence, ethnicity, region and wealth index), experience of child mortality, fertility desire, parity (Live birth in the; last 2 years). For the sociodemographic variables, maternal age was coded from the dataset as “35–39”, “40–44”, “45–49” years, respectively. The highest level of education was recoded as “No education”, “Primary”, “Secondary,” and “Higher”. Marital Status was recoded as “Never in Union”, Formerly in Union” and “Currently in Union”. Place of residence was coded “Urban” and “Rural”. Ethnicity was recoded as “Hausa”, “Igbo”, “Yoruba” and “Others”. Region was coded as “North Central”, North East, “North West”, “South West”, “South East” and “South South”, Wealth Index was recoded as “Poor”, “Middle” and “Rich”. Experience of child mortality was also recoded as “Ever experienced” and “Never experienced”. More so, Parity was measured as “No parity” and “One or More parity”. Media exposure was derived from the frequency of reading newspapers, frequency of listening to radio and frequency of watching TV.

### Data analysis

All analyses in the study were performed using Stata version 17. Sample socio-demographic characteristics were described using frequency distribution and percentage. A simple cross-tabulation was performed to obtain the percentage of use and non-use of contraceptives among the respondents. The multinomial logistic regression was applied for two purposes. Firstly, unadjusted multinomial logistic regression coefficients were applied to examine the separate bivariate relationship between use and non-use of contraceptive and the explanatory variables. Secondly, the relative risk ratios (rrr) were applied to examine the multivariate influence of the selected socio-demographic variables on the use and non-use of contraceptives.

The study employed the Stata version 17 statistical software for data analysis, which involved three phases: univariate, bivariate, and multivariate analysis. The socio-demographic characteristics of the respondents were examined to understand the study population and to organise and check the frequency distribution of respondents, weighted as specified. The dependent categorical variable was presented using bar charts. Bivariate analyses explored the association between Socio-demographic characteristics, other factors (such as media exposure, parity, experience of child mortality, fertility desire), and contraceptive use. The relationship was assessed using chi-square tests of association. At the multivariate level, multinomial logistic regression was employed, incorporating variables that demonstrated significance (*p* < 0.05) at the bivariate level.

### Ethical consideration

This study analysed secondary survey data from UNICEF, where all participants’ personally identifiable information had been removed. The National Statistical Office, the National Bureau of Statistics, and UNICEF obtained informed consent from survey participants before their participation. In addition, upon completing the registration process, the authors were granted permission to download and use the datasets. The data are available online: https://mics.unicef.org/surveys?page=4.

## Result

### Frequency distribution of socio-demographic factors and older factors of older reproductive women

Table 1 presents the socio-demographic characteristics of respondents. The total sample consists of 11,248 women, with the largest proportion (39.09%) in the 35–39 age group. The representation gradually declines with age, with 33.62% in the 40–44 bracket and 27.28% in the 45–49 group. Educational attainment among these women is relatively low, with a significant portion (30.84%) having no formal education. While 32.12% attained secondary education, only 15.58% have pursued higher education. The remaining 21.46% have completed only primary education. Also, marital status distribution indicates that the vast majority of these women (88.39%) are currently in a union, either married or cohabiting. Another 10.85% were formerly in a union, likely due to widowhood, divorce, or separation, while a mere 0.76% have never been in a union.

More so, the regional distribution of the population shows that women are spread across six main regions, with the highest representation in the South West (24.81%), followed by the North West (21.65%). The South-South region accounts for 16.00%, while the North-Central region represents 14.09%. The North East (10.67%) and South East (12.78%) have the lowest proportions. Ethnic diversity is notable in the sample, with the Hausa (21.81%), Yoruba (21.09%), and Igbo (17.74%) forming significant proportions of the population. However, a large proportion (39.36%) falls under the other ethnic group, reflecting the multi-ethnic nature of the country.

More so, the wealth index categories the women of advanced reproductive into three groups, with the largest proportion (47.15%) classified as been Rich. However, a considerable portion (34.07%) are classified as being poor, while 18.78% belong to the Middle Wealth Group. Regarding place of residence, the population is nearly evenly split, with 51.99% living in rural areas and 48.01% in urban areas.

For the other factors, the result also showed that the level of media exposure among respondents is predominantly low, with 65.89% falling into this category. A smaller proportion (27.90%) has moderate exposure, while only 6.22% of women report high media exposure. Considering parity in the last 2 years, a majority (78.71%) of the women did not have a live birth during this period, while 21.29% reported having one or more live births. The table also revealed that 31.88% of the respondents reported experiencing child mortality, while 68.12% had never lost a child. When asked about their fertility intentions, an overwhelming majority of respondents (94.22%) were undecided about having more children. Only 3.60% expressed a desire for more children, while 2.18% explicitly stated that they did not want more children.

**Table 1:** Frequency distribution of Socio-demographic characteristics and other factors of older reproductive women

Socio-demographic Characteristics	Frequency (*N* = 11,248)	Percentage (100%)
**Age Group**		
35–39	4,397	39.09
40–44	3,782	33.62
45–49	3,069	27.28
**Maternal Education**		
None	3,469	30.84
Primary	2,413	21.46
Secondary	3,613	32.12
Higher	1,753	15.58
**Marital Status**		
Never in union	85	0.76
Formerly in union	1,220	10.85
Currently in a union	9,943	88.39
**Region**		
North Central	1,584	14.09
North East	1,120	10.67
North West	2,435	21.65
South West	2,791	24.81
South West	1,437	12.78
South South	1,799	16.00
**Ethnicity**		
Hausa	2,453	21.81
Igbo	1,996	17.74
Yoruba	2,372	21.09
Others	4,427	39.36
**Wealth Index**		
Poor	3,833	34.07
Middle	2,112	18.78
Rich	5,303	47.15
**Place of Residence**		
Urban	5,340	48.01
Rural	5,848	51.99
**Other Factors**		
**Media Exposure**		
Low	7,411	65.89
Moderate	3,138	27.90
High	699	6.22
**Parity in the last 2 years**		
None	8,853	78.71
One or more	2,395	21.29
**Experience of child mortality**		
Never Experienced	7,662	68.12
Experienced	3,586	31.88
**Fertility Desire**		
Desire no children	245	2.18
Desire more children	405	3.60
Undecided	10,597	94.22

Source: Author analysis based on 2021 MICS Survey

### Prevalence of contraceptive methods use among older reproductive women

**Fig. 1:**
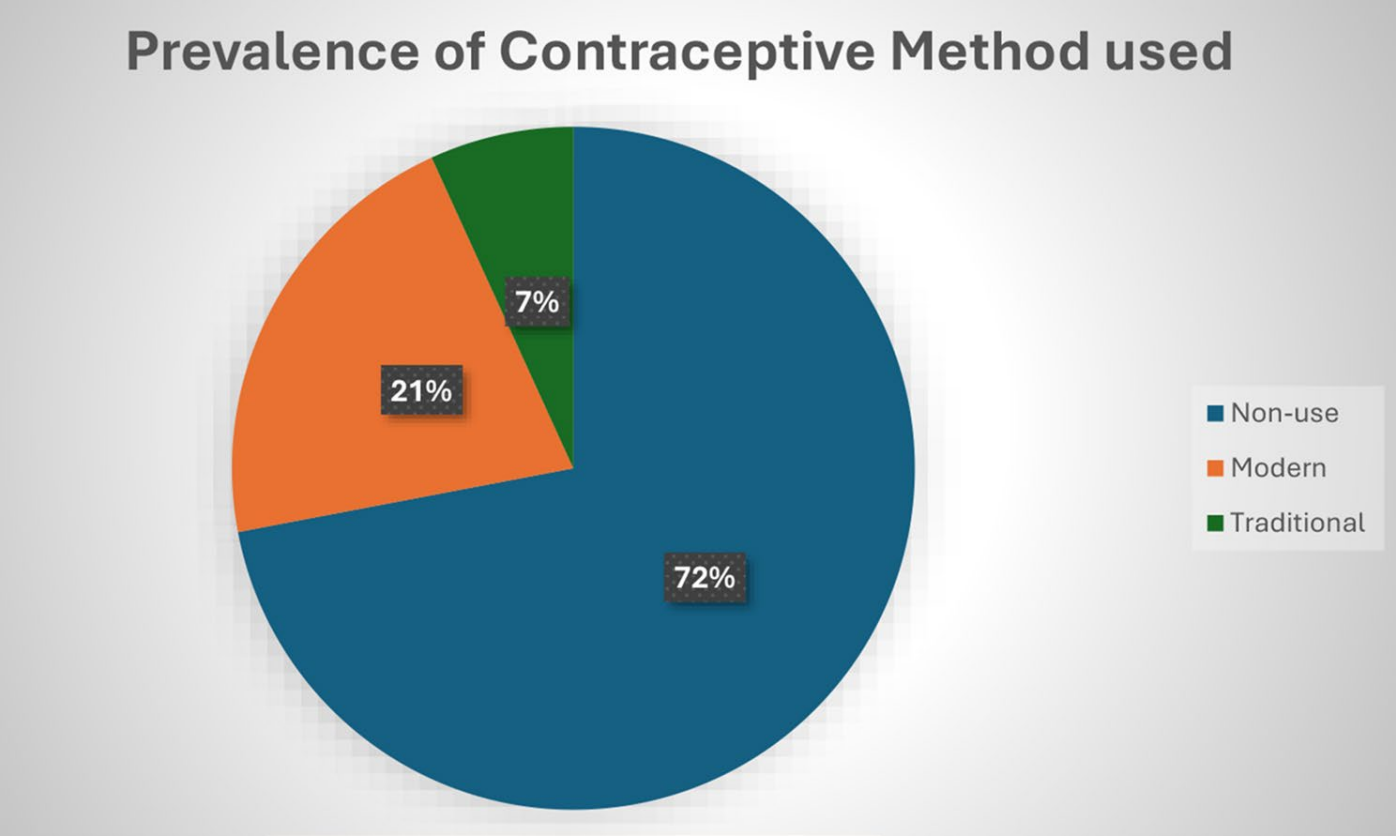
Showing the prevalence of Contraceptive use among Older reproductive women

The chart presented in Fig. 1 shows that 72% of older reproductive women do not use any method of contraception, 7% rely on traditional methods, while only 21% use modern methods.

## Bivariable analysis

### Association between socio-demographic characteristics, other factors, and contraceptive use among older reproductive women

Table 2 presents the association between socio-demographic characteristics, other factors, and contraceptive use among older reproductive women, based on data from the 2021 MICS Survey. Contraceptive use is categorised into three groups: non-use, use of modern methods, and use of traditional methods. The results show that all the socio-demographic variables assessed are significantly associated with contraceptive use, as indicated by their respective chi-square values and p-values (all p-values < 0.05).

The results showed that age is significantly associated with contraceptive use (χ² = 45.044, *p* = 0.002). The highest use of modern contraceptives was among women aged 35–39 where more than two-fifths (42.68) of them reported in the affirmative and over one-third (37.75%) also had a relatively high level of traditional method use. Maternal education shows a strong association with contraceptive use (χ² = 553.308, *p* < 0.001). Women with no formal education had the highest rate of non-use (36.49%), while those with secondary and higher education were more likely to use both modern (43.26% and 20.61%, respectively) and traditional methods (40.83% and 25.01%, respectively). More so, there is a significant association between marital status and contraceptive use with higher percentage showing non-use of any contraceptives (χ² = 80.072, *p* < 0.001).

Likewise, the results depicted that ethnicity plays an important role in contraceptive behaviour (χ² = 639.609, *p* < 0.001). Non-use of contraceptives is most prevalent among the Hausa ethnic group (25.32%), whereas modern and traditional method use is more common among the Yoruba and Igbo groups. The Igbos had the highest proportion of traditional method users (40.54%), while the Yoruba had the highest modern method use (31.19%). Region of residence is another strong predictor of contraceptive use (χ² = 657.272, *p* < 0.001). Women from the South West region reported the highest use of modern methods (36.77%) and traditional methods (31.12%), while non-use was more prevalent among women in the North West and North East regions. Similarly, wealth index is significantly associated with contraceptive use (χ² = 553.718, *p* < 0.001). Women in rich households had the highest levels of both modern (63.04%) and traditional (67.25%) contraceptive use, whereas non-use was most common among women from the poorest households (40.00%).

Place of residence is significantly related to contraceptive use (χ² = 354.274, *p* < 0.001). A large proportion of urban women are more likely to use contraceptives (62.06% use modern methods and 63.78% use traditional methods) compared to more than half (57.61%) of rural women who reported non-use of any methods.

More so, media exposure was a significant determinant of contraceptive use (χ² = 494.812, *p* < 0.001). Among women with low media exposure, a considerable majority (72.14%) reported non-use of contraception. In contrast, women with moderate and high levels of media exposure had higher proportions of modern contraceptive use (39.47% and 9.61%, respectively). A similar pattern was observed with traditional methods. Parity also showed a significant association with contraceptive use (χ² = 61.690, *p* < 0.001). Women who had not given birth in the last two years were more likely to use both modern (84.13%) and traditional (81.94%) methods compared to those with one or more children.

Similarly, experience of child mortality significantly influenced contraceptive use patterns among older reproductive women (χ² = 140.922, *p* < 0.001). Women who had never experienced the death of a child were more likely to use both modern (76.01%) and traditional (78.42%) contraceptive methods than those who had experienced child loss. More so, Fertility desire was also found to be significantly associated with contraceptive use (χ² = 16.668, *p* = 0.046). However, a substantial majority of respondents across all categories of contraceptive use reported being undecided about future fertility intentions (non-users: 95.75%, modern users: 94.99%, traditional users: 96.78%). Among the few women who expressed a desire not to have more children, the modern method use was slightly more common (1.58%) compared to those who wanted more children (3.43%).

**Table 2:** Association between Socio-demographic Characteristics, other factors, and contraceptive use among older reproductive women

Variables	Contraceptive Use			
Socio-demographic Characteristics	Non-use (8,100)	Modern (2,382)	Traditional (766)	Chi-square & *p*-value
**Age Group**				χ^**2**^ **=** 45.044
35–39	3,091 (36.16)	1,017 (42.68)	289 (37.75)	*P* = 0.002
40–44	2,664 (32.89)	836 (35.08)	282 (36.08)	
45–49	2,345 (28.95)	530 (22.23)	194 (25.39)	
**Maternal Education**				χ^**2**^ **=** 553.308
None	2,955 (36.49)	385 (16.17)	129 (16.80)	*P* < 0.001
Primary	1,805 (22.28)	476 (19.96)	133 (17.37)	
Secondary	2,270 (28.03)	1,030 (43.26)	313 (40.83)	
Higher	1,070 (13.20)	491 (20.61)	192 (25.01)	
**Marital Status**				χ^**2**^ **=** 80.072
Never in union	66 (0.82)	12 (0.49)	7 (0.95)	*P* < 0.001
Formerly in union	1,010 (12.48)	161 (6.76)	49 (6.37)	
Currently in union	7,023 (86.70)	2,209 (92.75)	710 (92.69)	
**Ethnicity**				χ^**2**^ **=** 639.609
Hausa	2,051 (25.32)	346 (14.55)	56 (7.37)	*P* < 0.001
Igbo	1,249 (15.41)	437 (18.33)	311 (40.54)	
Yoruba	1,422 (17.56)	767 (31.19)	183 (23.88)	
Others	3,378 (41.71)	832 (34.94)	216 (28.21)	
**Region**				χ^**2**^ **=** 657.272
North Central	1,108 (13.68)	387 (16.26)	89 (11.60)	*P* < 0.001
North East	1,029 (12.70)	154 (6.45)	17 (2.26)	
North West	1,996 (24.64)	382 (16.02)	58 (7.53)	
South West	1,676 (20.70)	876 (36.77)	238 (31.12)	
South East	953 (11.76)	255 (10.68)	231 (30.10)	
South South	1,337 (16.51)	329 (13.81)	133 (17.41)	
**Wealth Index**				χ^2^ = 553.718
Poor	3,240 (40.00)	463 (19.43)	130 (16.93)	*P* < 0.001
Middle	1,573 (19.42)	418 (17.54)	121 (15.82)	
Rich	3,286 (40.57)	1,502 (63.04)	515 (67.25)	
**Place of Residence**				χ^**2**^ **=** 354.274
Urban	3,433 (42.39)	1,478 (62.06)	489 (63.78)	*P* < 0.001
Rural	4,667 (57.61)	904 (37.94)	277 (36.22)	
**Other Factors**				
**Media Exposure**				χ^**2**^ **=** 494.812
Low	5,843 (72.14)	1,213 (50.92)	354 (46.27)	*p* < 0.001
Moderate	1,866 (23.04)	940 (39.47)	331 (43.23)	
High	390 (4.82)	229 (9.61)	80 (10.50)	
**Parity**				χ^**2**^ **=** 61.690
None	6,221 (76.81)	2,004 (84.13)	628 (81.94)	*P* < 0.001
One or More	1,879 (23.19)	378 (15.87)	138 (18.06)	
**Experience of Child Mortality**				χ^**2**^ **=** 140.922
Never Experienced	5,251 (64.83)	1,811 (76.01)	601 (78.42)	*P* < 0.001
Experienced	2,849 (35.17)	572 (23.99)	165 (21.58)	
**Fertility Desire**				χ^**2**^ **=** 16.668
Want no More	198 (2.45)	38 (1.58)	10 (1.23)	*P* = 0.046
Want More Children	308 (3.81)	82 (3.43)	15 (1.98)	
Undecided	7,593 (95.75)	2,263 (94.99)	741 (96.78)	

Source: Author analysis based on 2021 MICS Survey

## Multivariable analysis

### Multinomial logistic regression showing the influence of relative risk ratios on non-use of contraceptives with traditional method as the base outcome

Table 3 presents the relative risk ratios (RRRs) assessing the likelihood of non-use of contraceptives compared to the use of traditional methods, using multinomial logistic regression across three models.

In Model 1, which included only socio-demographic characteristics, education, ethnicity, region, and wealth status were significantly associated with contraceptive non-use. Women with primary education had a significantly higher risk of non-use compared to those with no education (RRR = 1.491; 95% CI: 0.999–2.226; *p* = 0.050). Ethnic differences were notable, as Igbo women were significantly less likely to report non-use relative to traditional use compared to Hausa women (RRR = 0.298; 95% CI: 0.145–0.609; *p* = 0.001). Regional disparities also emerged: women residing in the North East were over four times more likely to report non-use compared to those in the North Central region (RRR = 4.173; 95% CI: 2.412–7.222; *p* < 0.001), while those in the North West also had elevated odds (RRR = 2.137; 95% CI: 1.195–3.822; *p* = 0.010). Additionally, women in the rich wealth category were significantly less likely to report non-use compared to their poor counterparts (RRR = 0.568; 95% CI: 0.396–0.814; *p* = 0.002).

In Model 2, which focused on other factors, media exposure and experience of child mortality were significantly associated with contraceptive non-use. Compared to women with low media exposure, those with moderate media exposure had lower odds of non-use (RRR = 0.367; 95% CI: 0.257–0.525; *p* < 0.001), as did those with high exposure (RRR = 0.329; 95% CI: 0.188–0.577; *p* < 0.001). Furthermore, women who had experienced child mortality were more likely to report non-use relative to traditional method users (RRR = 1.614; 95% CI: 1.166–2.235; *p* = 0.004).

In the combined model (Model 3), several of these associations remained significant. Primary education continued to be associated with increased risk of non-use (RRR = 1.520; 95% CI: 1.009–2.290; *p* = 0.045). The reduced likelihood of non-use among Igbo women persisted (RRR = 0.293; 95% CI: 0.141–0.606; *p* = 0.001). Regionally, women from the North East (RRR = 3.989; 95% CI: 2.304–6.907; *p* < 0.001) and North West (RRR = 1.968; 95% CI: 1.101–3.517; *p* = 0.022) were significantly more likely to report non-use than those from the North Central region. Moderate media exposure continued to reduce the likelihood of non-use (RRR = 0.627; 95% CI: 0.409–0.961; *p* = 0.032), although the association for high exposure was not statistically significant in the full model.

**Table 3:** Relative risk ratios showing influence on Non-use of contraceptive with traditional method as base outcome

Factors	Model 1 (Socio-demographics)			Model 2 (Other Factors)			Model 3 (Combine effects of models 1 and 2)		
	RRR	*p*-value	95% CI	RRR	*p*-value	95% CI	RRR	*p*-value	95% CI
**Maternal Age**									
35–39 (ref)	**1.000**	**-**	**-**				**1.000**	**-**	**-**
40–44	0.827	0.197	0.620–1.103				0.844	0.261	0.628–1.135
45–49	1.001	0.991	0.779–1.288				1.007	0.960	0.768–1.320
**Education**									
None (ref)	**1.000**	**-**	**-**				**1.000**	**-**	**-**
Primary	1.491	0.050*	0.999–2.226				1.520	0.045*	1.009–2.290
Secondary	1.192	0.367	0.814–1.747				1.301	0.168	0.895–1.889
Higher	1.099	0.689	0.692–1.745				1.277	0.290	0.812–2.009
**Marital Status**									
Never in union (ref)	**1.000**	**-**	**-**				**1.000**	**-**	**-**
Formerly in union	2.187	0.199	0.662–7.224				2.289	0.168	0.705–7.431
Currently in union	0.894	0.846	0.286–2.792				0.932	0.903	0.303–2.869
**Ethnicity**									
Hausa (ref)	**1.000**	**-**	**-**				**1.000**	**-**	**-**
Igbo	0.298	0.001*	0.145–0.609				0.293	0.001*	0.141–0.606
Yoruba	0.564	0.105	0.282–1.127				0.559	0.101	0.279–1.120
Others	0.671	0.176	0.376–1.196				0.661	0.167	0.368–1.188
**Region**									
North Central (ref)	**1.000**	-	-				**1.000**	**-**	**-**
North East	4.173	< 0.001*	2.412–7.222				3.989	< 0.001*	2.304–6.907
North West	2.137	0.010*	1.195–3.822				1.968	0.022*	1.101–3.517
South West	0.836	0.517	0.487–1.436				0.863	0.594	0.503–1.482
South East	0.857	0.694	0.397–1.850				0.855	0.692	0.393–1.859
South South	0.917	0.692	0.598–1.408				0.893	0.609	0.578–1.379
**Wealth Index**									
Poor (ref)	**1.000**	-	-				**1.000**	**-**	**-**
Middle	0.764	0.137	0.535–1.089				0.799	0.222	0.558–1.145
Rich	0.568	0.002*	0.396–0.814				0.702	0.093	0.465–1.061
**Place of Residence**									
Urban (ref)	**1.000**	-	-				**1.000**	**-**	**-**
Rural	1.100	0.583	0.782–1.547				1.075	0.684	0.758–1.526
**Media Exposure**									
Low (ref)				**1.000**	**-**	**-**	**1.000**	**-**	**-**
Moderate				0.367	< 0.001	0.257–0.525	0.627	0.032*	0.409–0.961
High				0.329	< 0.001	0.188–0.577	0.658	0.239	0.328–1.322
**Parity**									
None (ref)				**1.000**	-	-	**1.000**	**-**	**-**
One or more live births				1.106	0.571	0.782–1.564	1.024	0.894	0.719–1.458
**Experience of child mortality**									
Never experienced (ref)				**1.000**	-	-	**1.000**	**-**	**-**
Ever experienced				1.614	0.004*	1.166–2.235	1.171	0.270	0.885–1.551
**Fertility desire**									
No more children (ref)				**1.000**	-	-	**1.000**	**-**	**-**
Want more children				1.072	0.893	0.385–2.986	1.414	0.541	0.465–4.298
Undecided				0.604	0.233	0.233–1.383	0.716	0.289	0.289–1.766

p value less than 0.05 indicate significant; ref: reference category; RRR: Relative risk ratio

### Multinomial logistic regression showing the influence of relative risk ratios on modern contraceptive use with traditional method as base outcome

Table 4 presents the relative risk ratios (RRR) from multinomial logistic regression assessing the likelihood of modern contraceptive use relative to traditional method use among women of advanced reproductive age in Nigeria. Results are shown for socio-demographic factors (Model 1), other factors (Model 2), and the combined model (Model 3).

In Model 1, educational attainment emerged as a strong predictor of modern contraceptive use. Compared to women with no education, those with primary education were 2.58 times more likely to use modern contraceptives (RRR = 2.581; 95% CI: 1.672–3.985; *p* < 0.001), and those with secondary education had an even higher likelihood (RRR = 3.007; 95% CI: 1.973–4.582; *p* < 0.001). Similarly, women with higher education were significantly more likely to use modern methods (RRR = 2.374; 95% CI: 1.476–3.819; *p* < 0.001). The region also played a role; women from the North East (RRR = 2.664; 95% CI: 1.506–4.713; *p* = 0.001) were significantly more likely to use modern contraceptives compared to those in the North Central region. Conversely, women in the South East (RRR = 0.289; 95% CI: 0.136–0.615; *p* = 0.001) and South-South (RRR = 0.448; 95% CI: 0.289–0.693; *p* < 0.001) were significantly less likely to use modern contraceptives relative to traditional methods.

In Model 3, which integrated both socio-demographic and other factors, the association between education and modern contraceptive use remained strong and statistically significant. Women with primary education (RRR = 2.583; 95% CI: 1.653–4.036; *p* < 0.001), secondary education (RRR = 3.083; 95% CI: 2.037–4.667; *p* < 0.001), and higher education (RRR = 2.430; 95% CI: 1.505–3.924; *p* < 0.001) were all significantly more likely to use modern contraceptives compared to uneducated women. Regional variations persisted; the North East (RRR = 2.702; 95% CI: 1.528–4.779; *p* = 0.001) and North West (RRR = 1.876; 95% CI: 1.007–3.496; *p* = 0.048) showed higher likelihoods of modern contraceptive use relative to the North Central region, while the South East (RRR = 0.284; 95% CI: 0.131–0.614; *p* = 0.001) and South-South (RRR = 0.445; 95% CI: 0.285–0.695; *p* < 0.001) maintained significantly lower odds. Additionally, women aged 45–49 years were significantly less likely to use modern contraceptives compared to those aged 35–39 (RRR = 0.712; 95% CI: 0.517–0.979; *p* = 0.037). Lastly, parity was a significant factor; women with one or more live births were less likely to use modern contraceptives than nulliparous women (RRR = 0.586; 95% CI: 0.389–0.879; *p* = 0.010).

**Table 4:** Relative risk ratios showing influence on modern contraceptive use with traditional method as base outcome

Factors	Model 1 (Socio-demographics)			Model 2 (Other Factors)			Model 3 (Combine effects of models 1 and 2)		
	RRR	*p*-value	95% CI	RRR	*p*-value	95% CI	RRR	*p*-value	95% CI
**Maternal Age**									
35–39 (ref)	**1.000**	**-**	**-**				**1.000**	**-**	**-**
40–44	0.844	0.258	0.629–1.132				0.789	0.125	0.582–1.068
45–49	0.799	0.147	0.589–1.082				0.712	0.037*	0.517–0.979
**Maternal Education**									
None (ref)	**1.000**	**-**	**-**				**1.000**	**-**	**-**
Primary	2.581	< 0.001*	1.672–3.985				2.583	< 0.001*	1.653–4.036
Secondary	3.007	< 0.001*	1.973–4.582				3.083	< 0.001*	2.037–4.667
Higher	2.374	< 0.001*	1.476–3.819				2.430	< 0.001*	1.505–3.924
**Marital Status**									
Never in union (ref)	**1.000**	**-**	**-**				**1.000**	**-**	**-**
Formerly in union	2.048	0.294	0.537–7.815				2.180	0.253	0.572–8.312
Currently in union	1.811	0.342	0.532–6.159				2.007	1.264	0.591–6.819
**Ethnicity**									
Hausa (ref)	**1.000**	**-**	**-**				**1.000**	**-**	**-**
Igbo	0.554	0.137	0.254–1.207				0.551	0.140	0.249–1.216
Yoruba	0.989	0.978	0.467–2.097				0.969	0.937	0.455–2.069
Others	0.899	0.723	0.502–1.613				0.883	0.681	0.488–1.597
**Region**									
North Central (ref)	**1.000**	-	-				**1.000**	**-**	**-**
North East	2.664	0.001*	1.506–4.713				2.702	0.001*	1.528–4.779
North West	1.842	0.051	0.996–3.399				1.876	0.048*	1.007–3.496
South West	0.643	0.110	0.374–1.105				0.643	0.113	0.372–1.110
South East	0.289	0.001*	0.136–0.615				0.284	0.001*	0.131–0.614
South South	0.448	< 0.001*	0.289–0.693				0.445	< 0.001*	0.285–0.695
**Wealth Index**									
Poor (ref)	**1.000**	-	-				**1.000**	**-**	**-**
Middle	1.137	0.503	0.780–1.657				1.152	0.465	0.788–1.685
Rich	1.045	0.824	0.709–1.538				1.095	0.694	0.695–1.727
**Place of Residence**									
Urban (ref)	**1.000**	-	-				**1.000**	**-**	**-**
Rural	0.978	0.898	0.691–1.384				0.971	0.871	0.679–1.389
**Media Exposure**									
Low (ref)				**1.000**	-	-	**1.000**	**-**	**-**
Moderate				0.834	0.380	0.559–1.248	0.867	0.575	0.526–1.429
High				0.842	0.560	0.471–1.503	0.976	0.945	0.481–1.977
**Parity**									
None (ref)				**1.000**	-	-	**1.000**	**-**	**-**
One or more live births				0.699	0.072	0.474–1.033	0.586	0.010*	0.389-879
**Experience of child mortality**									
Never experienced (ref)				**1.000**	-	-	**1.000**	**-**	**-**
Ever experienced				1.122	0.498	0.805–1.564	1.028	0.852	0.766–1.380
**Fertility desire**									
No more children (ref)				**1.000**	-	-	**1.000**	**-**	**-**
Want more children				1.375	0.577	0.448–4.222	1.347	0.472	0.471–5.081
Undecided				0.576	0.239	0.230–1.442	0.634	0.361	0.238–1.688

p value less than 0.05 indicates significance; ref: reference category; RRR: Relative risk ratio

## Discussion of findings

This study critically examined the prevalence and determinants of contraceptive use and non-use among women of advanced reproductive age (35–49 years) in Nigeria, utilizing the 2021 Multiple Indicator Cluster Survey (MICS), a comprehensive and nationally representative dataset. Unlike much of the existing literature which tends to treat women aged 15–49 as a homogenous group, this study’s unique focus on older reproductive women provides essential age-disaggregated insights into fertility behaviour, an area often overlooked in both demographic research and policy interventions.

The socio-demographic profile of the respondents revealed stark disparities that shape contraceptive behavior in this age group. Women aged 35–39 made up the largest cohort, and a significant proportion had low educational attainment, resided in rural areas, and belonged to lower wealth quintiles. These characteristics serve as structural determinants of health that either constrain or enable reproductive choices. Education, for instance, emerged as a critical predictor of contraceptive use. Women with secondary or higher education were significantly more likely to use modern methods than their uneducated counterparts. This supports the findings by Apanga et al. (2020) and Ahinkorah et al. (2021), who argue that education fosters not only knowledge acquisition but also autonomy and negotiation power within relationships, a necessary condition for contraceptive uptake [2, 4].

The study also uncovered considerable regional and ethnic disparities. Non-use was most prevalent in the North East and North West regions, predominantly occupied by Hausa ethnic groups, whereas modern contraceptive use was higher among Yoruba and Igbo women, particularly in the South West. These findings align with OlaOlorun et al. (2020), who noted that cultural and religious barriers in Northern Nigeria continue to perpetuate low family planning uptake. Such regional inequalities suggest the persistence of systemic and deeply embedded norms that affect access, demand, and acceptability of contraceptive services [10].

The study also showed that the role of media exposure was particularly striking. Women with moderate to high exposure to mass media were significantly more likely to use modern contraceptive methods. This finding supports the Health Belief Model, which posits that cues to action, such as health messages from trusted media sources, increase the likelihood of adopting a healthy behavior [18]. In contrast, women with low media exposure reported the highest rates of non-use. This highlights the potential of strategic communication in reshaping fertility intentions and dismantling myths about contraceptive use, especially among older reproductive women whose fertility decisions are often shaped by generational norms and misinformation [5]. Furthermore, reproductive history and fertility desire also influenced contraceptive choices. Women who had not experienced recent births or child mortality were more likely to use contraception. Yet over 94% of respondents remained undecided about future childbearing, a finding that introduces a new dimension to the discourse: *fertility ambivalence*. According to [11], ambivalence in fertility desires among older reproductive women is often under-researched, yet it plays a critical role in contraceptive decision-making. The assumption that older reproductive women no longer desire children is flawed and contributes to programmatic invisibility [11].

More so, the multinomial logistic regression models provided a more granular understanding of how different factors jointly influence contraceptive use. Education remained a strong and statistically significant predictor across all models. Women with secondary or higher education were more than twice as likely to use modern contraceptives compared to those with no education (*p* < 0.001). Notably, even within regions where overall use was low, educated women were disproportionately represented among modern users. This indicates that education may mitigate some of the regional and cultural barriers that are being experienced by the women. Particularly, education is a very powerful tool in the Southern regions compared to the Northern regions, and this is because of the level of importance placed on education in the former compared to the latter which alters the cultural set-up of the women. Also, education affects the psychology and attitudes of women, hence, utilization of contraceptives can be influenced by the level of information and exposure that a woman has, which is basically informed by education [19]. Another noteworthy finding was that parity had a counterintuitive relationship with contraceptive use. Women with one or more children were less likely to use modern contraceptives compared to those with no children. This contradicts the commonly held assumption that parity corresponds with greater contraceptive need. As noted by Kundu et al. (2022), some high-parity women may adopt fatalistic attitudes toward childbearing or face social pressure to continue expanding their families until a desired composition is achieved, particularly male preference [16].

### Strength of the study

This study leveraged the Multiple Indicator Cluster Survey (MICS) dataset, a robust, nationally representative household survey employing a stratified cluster sampling technique. By harnessing this large-scale national survey, the research provides a dependable source of information on reproductive healthcare services, encompassing women’s utilization and non-utilization of contraceptives. The MICS dataset is acclaimed for its rigorous methodology and representative sampling, enhancing the study’s generalizability and reliability.

The study’s focus on regional variations across six geo-political zones offers nuanced insights into the complex dynamics of each region and their health outcomes. By examining regional variations in prevalence and factors associated with utilization and non-utilization of contraceptive use, the study addresses a crucial aspect of women’s reproductive health life. This approach contributes to knowledge and makes informed data relevant for policy-making in respect of this significant public health concern.

The study’s methodological adeptness is substantiated through the analysis of secondary data using appropriate statistical methods. Furthermore, the findings are policy-relevant, aligning with Nigeria’s development goals and global health initiatives, such as the Sustainable Development Goals (SDGs). The study’s results provide valuable evidence for policymakers and stakeholders, informing policy-making and intervention strategies tailored to specific regional needs.

Overall, this study adds to the growing body of research on women of reproductive ages and reproductive healthcare services utilization and non-utilization, particularly in the Nigerian context. The findings have significant implications for policy and practice, highlighting the need for targeted interventions to address regional prevalence and factors in reproductive healthcare services utilization and non-utilization. By providing a comprehensive understanding of the factors influencing reproductive healthcare services utilization and non-utilization, the study contributes to the development of effective strategies to improve maternal health outcomes in Nigeria.

### Limitations of the study

Notwithstanding the study’s methodological strengths, several limitations warrant consideration. The reliance on secondary data from the Multiple Indicator Cluster Survey (MICS) dataset constrains the study’s capacity for granular control over variables and measurement instruments. The predefined survey questions and response categories may not comprehensively capture the nuances of regional variations and reproductive healthcare services utilization and non-utilization, potentially introducing measurement biases that could compromise the accuracy of the findings. The study’s generalizability is also circumscribed by its exclusive focus on the Nigerian context, which may limit the applicability of the findings to other cultural or geographical settings. Consequently, caution should be exercised when extrapolating the results to diverse populations or environments, highlighting the need for replication studies in other contexts to enhance the external validity of the research. Moreover, future studies could benefit from more nuanced and context-specific data collection instruments, as well as longitudinal designs that facilitate the examination of temporal relationships and causal pathways in reproductive healthcare services utilization and non-utilization.

## Conclusion

This study underscores the persistent and multifaceted challenges affecting contraceptive uptake among women of advanced reproductive age in Nigeria. Despite national and global efforts to improve access to family planning, a significant proportion of older reproductive women remain unreached due to intersecting socio-demographic, cultural, and systemic barriers. The findings reveal that education, ethnicity, regional location, wealth, and media exposure are central to shaping contraceptive behavior. Notably, the high level of fertility ambivalence signals a neglected area in reproductive health programming.

A one-size-fits-all approach to family planning is insufficient. There is an urgent need to rethink how older reproductive women are approached in reproductive health discourse, not as a demographic that has outlived its reproductive relevance, but as one that is uniquely positioned at the intersection of risk and unmet need. As Nigeria continues to pursue its FP2030 commitments and the Sustainable Development Goals (SDGs), targeted investment in this age group is essential to reduce maternal mortality, and unintended pregnancies, and foster reproductive autonomy.

## Recommendations

In light of the findings from this study, several strategic recommendations are proposed to enhance reproductive health services and contraceptive uptake among women in Nigeria. National reproductive health policies must prioritize age-specific fertility counseling, particularly for women aged 35 to 49. These policies must address the unique experiences of reproductive ambivalence and uncertainty that often characterize this age group. Institutionalizing such counseling strategies will ensure that older reproductive women receive accurate information and empathetic support tailored to their reproductive needs. Furthermore, there is a critical need to strengthen community education and advocacy programs aimed at dismantling deeply rooted cultural and social norms that hinder contraceptive use among older reproductive women. These interventions should be continuous and inclusive, engaging both women and men while being sensitive to the religious and cultural dynamics prevalent in various regions. Sustained efforts in public education can foster a more supportive environment for informed reproductive decision-making.

In addition, expanding access to family planning information through media and technology presents a viable and scalable solution. Platforms such as radio, mobile applications, and social media should be strategically employed to disseminate accurate, relatable, and culturally appropriate information on contraceptive options for older reproductive women. These channels can bridge existing knowledge gaps and reach diverse audiences, especially in underserved areas.

Equally important is the training of healthcare providers in delivering non-discriminatory and inclusive contraceptive counseling. Health workers must be equipped with the skills and sensitivity required to provide respectful care that does not marginalize older reproductive women or make assumptions about their reproductive choices. Such training will contribute to a more equitable and person-centered family planning service landscape.

Integrating family planning into routine health services also shows promise. Contraceptive counseling should be incorporated within broader women’s health checks, including cervical cancer screenings and consultations related to menopause. This integration will help normalize discussions about contraception for older reproductive women and present it as a standard aspect of overall health and wellness. Finally, future research should explore the influence of male partners and prevailing gender norms on contraceptive decisions among older Nigerian women. A deeper understanding of spousal dynamics, gender roles, and the impact of male approval can inform more comprehensive and effective family planning strategies. By implementing these multidimensional recommendations, Nigeria can progress toward a more inclusive reproductive health framework that respects the needs and rights of women at all stages of their reproductive lives. The reproductive journey does not end at 35; rather, it becomes more complex and necessitates a more refined understanding, support, and respect from both policy and practice.

## Data Availability

Secondary data (women’s dataset) analysed can be accessed online at [https://mics.unicef.org/surveys?page=4] (https://mics.unicef.org/surveys?page=4).

## Abbreviations

EAs: Enumeration areas
MICS: Multiple Indicator Cluster Survey
PSUs: Primary sampling units
SSS: sub-Saharan Africa
NDHS: National Demographic and Health Survey
RRR: Relative Risk Ratios
UNICEF: United Nations Population Funds
